# Involvement of Aberrant Glycosylation in Thyroid Cancer

**DOI:** 10.1155/2010/816595

**Published:** 2010-06-27

**Authors:** Eiji Miyoshi, Yasuhiro Ito, Yoko Miyoshi

**Affiliations:** ^1^Department of Molecular Biochemistry and Clinical Investigation, Graduate School Medicine, Osaka University, 1-7 Yamada-oka Suita 565-0871, Japan; ^2^Department of Surgery, Kuma Hospital, 8-2-35, Shimoyamate-dori, Chuo-ku, Kobe 650-0011, Japan; ^3^Department of Pediatrics, Graduate School Medicine, Osaka University, 2-2 Yamada-oka, Suita 565-0871, Japan

## Abstract

Glycosylation is one of the most common posttranslational modification reactions and nearly half of all known proteins in eukaryotes are glycosylated. In fact, changes in oligosaccharides structures are associated with many physiological and pathological events, including cell growth, migration and differentiation, and tumor invasion. Therefore, functional glycomics, which is a comprehensive study of the structures and functions of glycans, is attracting the increasing attention of scientists in various fields of life science. In cases of thyroid cancer, the biological characters and prognosis are completely different in each type of histopathology, and their oligosaccharide structures as well as the expression of glycosyltransferases are also different. In this review, we summarized our previous papers on oligosaccharides and thyroid cancers and discussed a possible function of oligosaccharides in the carcinogenesis in thyroid cancer.

## 1. Introduction

Oligosaccharides are mostly found on the cell surface and extracellular matrix (ECM), and also in various organelles such as the Golgi, ER, lysosome, cytosol, and nuclei. As compared to research on DNA, RNA, and proteins, study on glycans is rather difficult and the research in this field has been neglected for a long period, the same being true for glycomics as compared to proteomic research. In order to characterize the structures of glycans, glycobiology including glycomics is essential for understanding of the structures and functions of proteins. In the last couple of years, most of the glycosyltransferases (over 180 glycosyltransferase genes) have been identified, based on the genome sequence data and bioinformatics approach. Glycosylation reactions are catalyzed by the actions of glycosyltransferases; sugar chains are added to various complex carbohydrates. Modified oligosaccharides have the ability to interfere with carbohydrate-protein or protein/glycoprotein-glycoprotein interactions and, as a result, regulate many physiological and pathological events, including cell growth, migration and differentiation, and tumor metastasis. Cell surface carbohydrates contribute to a variety of interactions between a cell and its extracellular environment, since they are located on the outermost layer of the cell. Carbohydrates are the first molecules to be encountered and recognized by other cells, antibodies, invading viruses, and bacteria. Many secreted molecules such as hormones and toxins have also been reported to bind to carbohydrate receptors on the cell surface. Most receptors on the cell surface are *N*-glycosylated, including epithelial growth factor receptor (EGFR), integrins, and transforming growth factor *β* receptor (TGF*β* R). Increasing evidence indicates that sugar chains on glycoproteins are involved in the regulation of cell-cell communication, signal transduction, and protein folding and stability [2–5]. During the carcinogenesis in thyroid cancer, the biological characteristics of tumor cells dramatically changed with malignant transformation into undifferentiated thyroid cancer. In this period, the expression of glycosyltransferases as well as their target proteins would also be altered. The alteration of oligosaccharide structures on the cell surface could control cellular differentiation and biological characteristics. Also, aberrant expression of glyco-related genes could be a marker of thyroid cancer. In this review, we mainly focus on the importance of oligosaccharides in thyroid cancer, according to our previous reports.

## 2. Why Did We Start a Glycomics Project?

**Figure 1:**
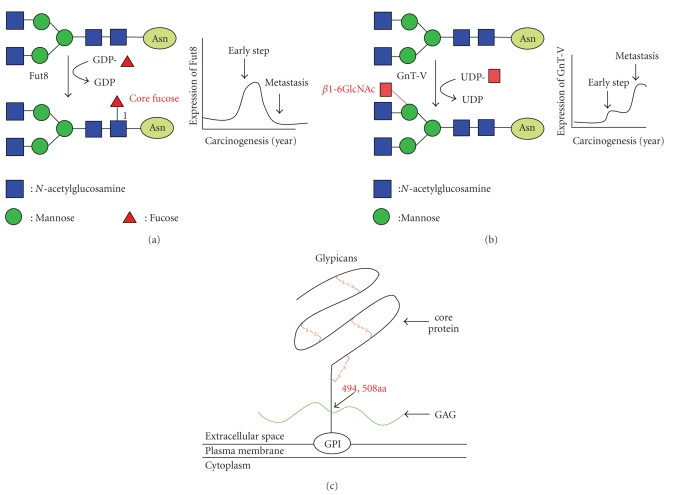
Oligosaccharide-related molecules in this review. (a) Reaction pathway catalyzed by Fut8. Expression of Fut8 was increased at the early phase of carcinogenesis and decreased at the advanced stage. (b) Reaction pathway catalyzed by GnT-V. Expression of GnT-V was up-regulated in a two-step manner. (c) Structure of GPC3. GPC 3 is a proteoglycan with two oligosaccharides at the 494 and 508 amino acid residues and binds to cell membranes through a GPI-anchor. GPC3-deficient mice showed overgrowth a14-day embryo stage.

Glycobiology is one of the most difficult research areas in life science because oligosaccharides exhibit a variety of aspects and their functions often differ with the organ, species, or type of cancer. In terms of cancer, prominent changes in oligosaccharide structures on glycoproteins are dependent on sialylation, fucosylation, and branching formation. Both sialylation and fucosylation modify the charges on total oligosaccharide structures and thus controll receptor and adhesion molecules on the cell surface. Sialylation of IgG oligosaccharides can regulate allergic reactions and this minor IgG group suppresses autoimmuno reactions [6, 7]. Fucosylation is one of the most important oligosaccharide modifications, being linked to cancer and inflammation [8]. Many reports have implied the involvement of branching formation of *N*-glycans and * N*-acetylglucosaminyltransferase V (GnT-V) which is key enzymes producing the branching [9]. Regarding many biological phenomena, Dr Taniguchi's group have succeeded in the purification and characterization of glycosyltransferases, which are involved in the branching formation for N-glycans [10]. Involving cDNAs or antibodies for these glycosyltransferases, many studies have revealed the relationship to carcinogenesis and/or tumor metastasis. Figure 1 shows three targets of glyco-related proteins in this review. Basically, the expression of Fut8 and GnT-V is relatively low and increases with malignant transformation. Expression of Fut8 increases in the early phase of carcinogenesis, but decreases at the stage of metastasis. In contrast, two-step increases in GnT-V expression are observed in certain kinds of cancer. Expression of glypican 3, which is a proteoglycan belonging to the glypican family, increases/decreases in an organ-specific manner. 

## 3. Fut8 and Thyroid Cancer

**Figure 2:**
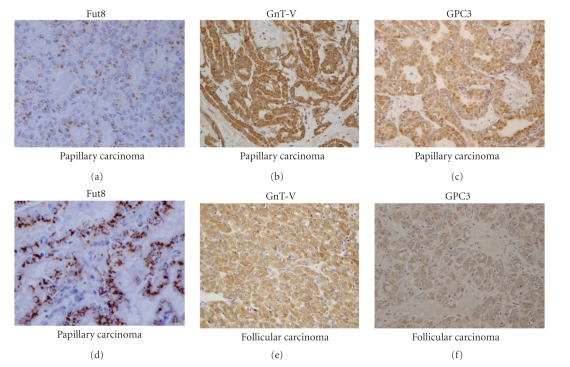
Immunohistochemical studies on Fut8, GnT-V, and GPC3 in thyroid cancer. The staining of Fut8 was located in the Golgi apparatus, while GnT-V staining was observed throughout a cell. Staining of GPC3 was heterogeneous and showed a membrane pattern.

 Fucosylation is one of the most important modes of glycosylation in cancer. Fucosylation is regulated by several kinds of fucosyltransferases, the GDP-fucose synthetic pathway and GDP-fucose transporters. Before these complicated mechanisms of fucosylation were clarified, fucosylated target proteins were found and used as tumor markers. Increases in fucosylated alpha-fetoprotein (AFP) were reported by Drs Breborowicz et al. [11] and Dr Taketa et al. [12]. They first found microheterogeneity of AFP in several liver conditions and then found increases in *α*1-6 fucosylation (core-fucosylation) of AFP on lectin affino-electrophoresis. AFP is a well-known tumor marker for hepatocellular carcinomas (HCCs), but it is sometimes also increased in benign liver diseases such as chronic hepatitis and liver cirrhosis. In contrast, AFP with core-fucosylation is a very specific marker of HCCs [13, 14]. AFP with core-fucosylation was called AFP-L3, because it was detected in the L3 fraction on LCA (Lens culinaris agglutinin) lectin-electorophoresis. Core-fucosylation comprises the attachment of fucose to the innermost *N*-acetylglucosamine in *N*-glycans. *α*1-6 fucosyltransferase (Fut8) catalyzes this core-fucosylation reaction. Dr. Uozumi et al. succeeded in the purification and cDNA cloning of Fut8 from porcine brain [15]. Studies on cancer and fucosylation have moved to the second stage since that time. Fut8 knockout mice show emphysema-like lesions in the lungs because core-fucose is important for the functions of reactions such as EGF-R and TGF*β*-R [5, 16]. When we established a monoclonal antibody for Fut8, we performed the first immunohistochemical study on thyroid cancer [17]. This is because there are various types of thyroid cancer and the prognosis differs for each pathological condition. As a result of immunohistochemical studies, involving 133 cases of thyroid cancer, the expression of Fut8 was found to be quite low in normal follicles. As shown in Figure 2, positive staining of Fut8 was observed in papillary carcinomas. This staining pattern could be called Golgi localization. The number of Fut8-positive cases of follicular carcinomas was relatively low. We concluded that expression of Fut8 might be a key factor for the progression of thyroid papillary carcinomas, but not of follicular carcinomas. High expression of Fut8 was observed in 33.3% of papillary carcinomas and the incidence was directly linked to tumor size and lymph node metastasis. In contrast, this phenomenon was less frequently observed in cases of follicular carcinomas and anaplastic (undifferentiated) carcinomas. Furthermore, decreases in Fut8 expression in papillary carcinomas might be linked to anaplastic transformation. These results seem to be similar to the results in the case of colon carcinogenesis that is, “fucosylation and defucosylation” [18]. Further studies should determine the biological function of core-fucose in thyroid cancer.

## 4. GnT-V and Thyroid Cancer

*N*-Acetylglucosaminyltransferase V (GnT-V) is one of the most important glycosyltransferases in tumor metastasis [9]. So far, more than 80 papers on GnT-V and tumor metastasis have appeared, with this number being quite high as compared to other glycosyltransferases. One mechanism underlying GnT-V and tumor progression is beta1-6 GlcNAc branching formation, a product of GnT-V, up-regulating cell surface growth factor receptors such as EGF-R [19]. The other mechanism is suppressing of the degradation of glycoproteins, resulting in an increase in protease expression [20]. Matriptase is a tumor-associated type II transmembrane serine protease that positively regulates carcinoma metastasis by activating the latent forms of hepatocyte growth factor (HGF) and urokinase-type plasminogen activator (uPA) [21]. The overexpression of GnT-V in gastric carcinoma cells enhances the degradation of matriptase and accelerated the peritoneal dissemination of these cancer cells in athymic mice [20]. Matriptase purified from GnT-V-transfected gastric cancer cells is resistant to trypsin and this resistance is dependent on the oligosaccharides linked to the 772 Asn residue of matriptase [22]. These findings indicate that GnT-V modifies the oligosaccharide structure of matriptase, thus altering the function of proteases. According to the results of these *in vitro* studies on GnT-V and matriptase, we performed immunohistochemical studies, using 132 cases of thyroid cancers [23]. While neither GnT-V nor matriptase was expressed in normal thyroid tissue, positive staining for matriptase and GnT-V was observed in 52/68 and 66/68 cases of papillary carcinomas, 3/23 and 10/23 cases of follicular carcinomas, 5/13 and 9/13 cases of follicular adenomas, and 11/28 and 6/28 cases of anaplastic carcinomas, respectively. Immunohistochemistry, as well as Western blotting, showed that the expression of matriptase paralleled the expression of GnT-V. However, the expression of matriptase mRNA was not correlated with its protein level, suggesting that the enhancement of matriptase expression could be caused by a posttranslational modification such as glycosylation through GnT-V-mediated glycosylation. In the case of papillary carcinomas, the levels of expression of both GnT-V and matriptase were significantly higher in tumors of 1 cm or less in size (microcarcinomas) and in cases without poorly differentiated lesions, and the two proteins were significantly correlated. In contrast, the prognosis of thyroid carcinomas after surgery was correlated with the expression of neither GnT-V nor matriptase, because the levels of their expression were quite low in anaplastic (undifferentiated) carcinomas. These results suggest that prolonged stabilization of matriptase is stabilized through GnT-V-mediated glycosylation *in vivo*, thus extending its halftime and permitting it to play a role in the early phases of papillary carcinomas, but not in the later phase of their progression. The staining pattern of GnT-V was different from that of Fut8 although GnT-V and Fut8 are glycosyltransferases located on the Golgi apparatus. GnT-V is cleaved by gamma secretase, resulting in its secretion into the serum or conditioned medium [24]. The secreted type of GnT-V acts as an angiogenesis cofactor [25].

## 5. Glypican 3 and Thyroid Cancer

**Figure 3:**
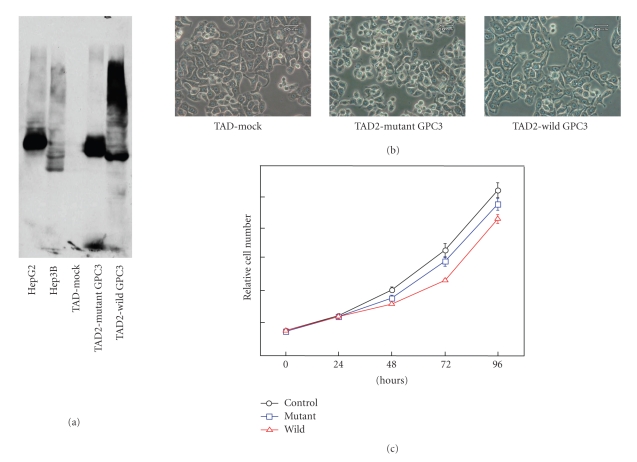
The effect of overexpression of GPC3 with/without oligosaccharides on thyroid cancer cell lines. Western blot analysis of GPC3 was performed using cellular proteins derived from HepG2 cells, Hep3B cells, parental TAD2 cells, mutant GPC3-transfected TAD2 cells, and wild type GPC3-transfected TAD2 cells. (b) Microscopic observation of parental TAD2 cells, wild type GPC3-transfected TAD2 cells, and mutant GPC3-transfected TAD2 cells. Morphological changes were observed for wild type GPC3-transfected TAD2 cells, as compared to the other two types of cells. (c) Cell proliferation was evaluated by means of WST-1 assaying.

Glypican 3 (GPC3) is one of the heparan sulfate proteoglycans (HSPGs) that are attached to the cell surface through a glycosylphosphatidylinositol (GPI) anchor [26]. While high expression of GPC3 is observed in fetal organs, it is scarcely detected in adult tissues. Interestingly, the serum levels of GPC3 measured by means of an enzyme-linked immunosorbent assay were increased in patients with HCC at 40 ~ 53%. Since there was no correlation between the GPC3 and alpha-feto protein levels, 82% of HCC patients were positive for at least 1 of these 2 tumor-markers. GPC3 was also identified as a tumor marker for melanomas [27]. GPC3 is involved in several kinds of cell signaling such as Wnt/Wingless, Hedgehog, TGF-*β*, and fibroblast growth factor, resulting in the stimulation of HCC growth [28]. When we performed immunohistochemical analysis of GPC3 in thyroid cancer [29], GPC3 was scarcely expressed in normal thyroid glands, but was dramatically enhanced in certain types of cancers including 100% of follicular carcinomas (20/20 cases) and 70% of papillary carcinomas (48/69 cases). Expression of GPC3 in follicular carcinomas was significantly higher than that of follicular adenomas (*P  *< .0019). In contrast, no expression of GPC 3 was observed in 20 cases of anaplastic carcinomas. When expression of GPC3 was investigated in 69 cases of papillary carcinomas according to their clinical background, it was found to be expressed at an early stage. These data prompted us to perform experiments on transfection of GPC3 into thyroid cancer cell lines to determine the biological function of GPC3. When the GPC3 gene was transfected into a human thyroid cancer cell line, TAD2, cell growth was dramatically suppressed in the wild type of GPC3 transfectants, but not markedly suppressed in the GPC3 oligosaccharide mutants (Figure 3). This oligosaccharide of GPC3 is heparan sulfate and the mutants were established by point mutation of the amino acid sequences of GPC3 expression vectors. The cell morphology dramatically changed with the transfection of GPC3 with oligosaccharides but not GPC3 without oligosaccharides. In the case of HCCs, oligosaccharides of GPC3 were not linked to cell signaling or cell growth [28]. Further studies are required to characterize GPC3-mediated cell signaling in thyroid cancer. 

## 6. Perspective

**Figure 4:**
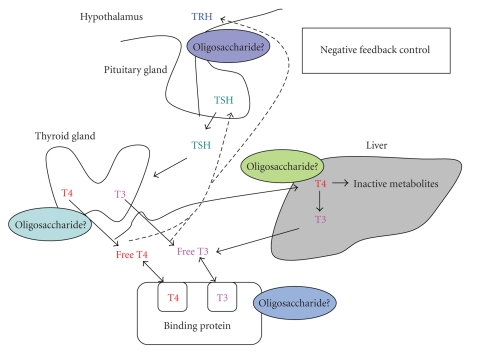
Oligosaccharides involve in the regulation of thyroid function. The production of thyroid hormone by the thyroid gland is regulated bythe hypothalamus and the pituitary gland. Hypothalamic thyrotropin-releasing hormone (TRH) induces the pituitary gland to release thyroid-stimulating hormone (TSH) into the general circulation, whereby it reaches the thyroid gland and stimulates the production and release of thyroid hormone [1].T4 is the predominant secretory product of the thyroid gland, with peripheraldeiodination of T4 to T3 in the liver and kidneys supplying roughly 80% of the circulating T3. Both circulating T3 and T4 directly inhibit TSH synthesis and are released independently; T4 via its rapid conversion to T3. Circulating T4 and T3 are bound predominantly to serum proteins. FT3 is the metabolically active form of thyroid hormone, whereas protein-bound T3 and T4 may be considered reservoirs of the hormone in equilibrium with the metabolically active free hormone.

The differential diagnosis of follicular adenocarcinoma from follicular adenomas is the most important issue. In certain cases, even pathological examination is not perfect. Galectin 3 has been reported in one of the candidate pathological markers for a differential diagnosis [30]. Recently, we established a quantitative ELISA assay for GPC3 and investigated its clinical usefulness for the diagnosis of thyroid cancer [31]. Galectin 3 binds to polylactosamine structures on N-glycans and controls the function of cell surface receptors. The polylactosamine structures are synthesized through the reaction of GnT-V and the molecular mechanisms underlying GnT-V-related cell signaling would be due to galectin 3 [19]. It is important to determine the biological function of galectin-3 in thyroid cancer. Basically, the thyroid is an endocrine organ that produces thyroid hormone. Many factors control the release of thyroid hormone via receptor-ligand mediated signaling (Figure 4). In this system, oligosaccharides seem to be deeply involved, and therefore control of thyroid function via oligosaccharides would be a promising research target. Many hormones including thyroid hormone could be regulated through glycosylation [32]. In this review, we did not describe sialic acids in thyroid carcinoma. Sialic acids are a terminal oligosaccharide on *N-/O*-glycans and have a variety of biological functions [33]. While the biological significance of sialic acids in thyroid cancer remains unknown, changes in cellular sialylation have been changed in malignant formation [34]. Transgenic or knockout mice as to glycosyltransferases including sialyltransferases would be a powerful tool for determining the oligosaccharide function in thyroid carcinogenesis directly. Such mice should be mated with model mice for thyroid cancer, if any.

## Abbreviations

Fut8: *α*1-6 fucosyltransferase
EGF-R: epithelial growth factor receptor
TGF*β* R: transforming growth factor *β* receptor
AFP: *α*-fetoprotein
HCC: hepatocellular carcinoma
(GnT-V): * N*-acetylglucosaminyltransferase V
GPC3: Glypican 3.

## References

[1] De Groot LJ *The Thyroid and Its Diseases*.

[2] Varki A (1993). Biological roles of oligosaccharides: all of the theories are correct. *Glycobiology*.

[3] Dwek RA (1995). Glycobiology: “Towards understanding the function of sugars”. *Biochemical Society Transactions*.

[4] Saxon E, Bertozzi CR (2001). Chemical and biological strategies for engineering cell surface glycosylation. *Annual Review of Cell and Developmental Biology*.

[5] Taniguchi N, Miyoshi E, Gu J, Honke K, Matsumoto A (2006). Decoding sugar functions by identifying target glycoproteins. *Current Opinion in Structural Biology*.

[6] Kaneko Y, Nimmerjahn F, Ravetch JV (2006). Anti-inflammatory activity of immunoglobulin G resulting from Fc sialylation. *Science*.

[7] Anthony RM, Nimmerjahn F, Ashline DJ, Reinhold VN, Paulson JC, Ravetch JV (2008). Recapitulation of IVIG anti-inflammatory activity with a recombinant IgG Fc. *Science*.

[8] Miyoshi E, Moriwaki K, Nakagawa T (2008). Biological function of fucosylation in cancer biology. *Journal of Biochemistry*.

[9] Dennis JW, Granovsky M, Warren CE (1999). Glycoprotein glycosylation and cancer progression. *Biochimica et Biophysica Acta*.

[10] Taniguchi N, Miyoshi E, Ko JH, Ikeda Y, Ihara Y (1999). Implication of *N*-acetylglucosaminyltransferases III and V in cancer: gene regulation and signaling mechanism. *Biochimica et Biophysica Acta*.

[11] Breborowicz J, Mackiewicz A, Breborowicz D (1981). Microheterogeneity of *α*-fetoprotein in patient serum as demonstrated by lectin affino-electrophoresis. *Scandinavian Journal of Immunology*.

[12] Taketa K, Izumi M, Ichikawa E (1983). Distinct molecular species of human *α*-fetoprotein due to differential affinities to lectins. *Annals of the New York Academy of Sciences*.

[13] Aoyagi Y, Suzuki Y, Igarashi K (1991). The usefulness of simultaneous determinations of glucosaminylation and fucosylation indices of alpha-fetoprotein in the differential diagnosis of neoplastic diseases of the liver. *Cancer*.

[14] Taketa K, Endo Y, Sekiya C (1993). A collaborative study for the evaluation of lectin-reactive *α*- fetoproteins in early detection of hepatocellular carcinoma. *Cancer Research*.

[15] Uozumi N, Yanagidani S, Miyoshi E (1996). Purification and cDNA cloning of porcine brain GDP-L-Fuc:*N*-acetyl-*β*-D-glucosaminide *α*1→6 fucosyltransferase. *Journal of Biological Chemistry*.

[16] Wang X, Inoue S, Gu J (2005). Dysregulation of TGF-*β*1 receptor activation leads to abnormal lung development and emphysema-like phenotype in core fucose-deficient mice. *Proceedings of the National Academy of Sciences of the United States of America*.

[17] Ito Y, Miyauchi A, Yoshida H (2003). Expression of *α*1, 6-fucosyltransferase (FUT8) in papillary carcinoma of the thyroid: its linkage to biological aggressiveness and anaplastic transformation. *Cancer Letters*.

[18] Moriwaki K, Noda K, Furukawa Y (2009). Deficiency of GMD leads to escape from NK cell-mediated tumor surveillance through modulation of TRAIL signaling. *Gastroenterology*.

[19] Lau KS, Partridge EA, Grigorian A (2007). Complex N-glycan number and degree of branching cooperate to regulate cell proliferation and differentiation. *Cell*.

[20] Ihara S, Miyoshi E, Ko JH (2002). Prometastatic effect of N-acetylglucosaminyltransferase V is due to modification and stabilization of active matriptase by adding *β*1-6 GlcNAc branching. *Journal of Biological Chemistry*.

[21] Lin C-Y, Anders J, Johnson M, Dickson RB (1999). Purification and characterization of a complex containing matriptase and a Kunitz-type serine protease inhibitor from human milk. *Journal of Biological Chemistry*.

[22] Ihara S, Miyoshi E, Nakahara S (2004). Addition of *β*1-6 GlcNAc branching to the oligosaccharide attached to Asn 772 in the serine protease domain of matriptase plays a pivotal role in its stability and resistance against trypsin. *Glycobiology*.

[23] Ito Y, Akinaga A, Yamanaka K (2006). Co-expression of matriptase and N-acetylglucosaminyltransferase V in thyroid cancer tissues; its possible role in prolonged stability in vivo by aberrant glycosylation. *Glycobiology*.

[24] Nakahara S, Saito T, Kondo N (2006). A secreted type of *β*1, 6 *N*-acetylglucosaminyltransferase V (GnT-V), a novel angiogenesis inducer, is regulated by *γ*-secretase. *FASEB Journal*.

[25] Saito T, Miyoshi E, Sasai K (2002). A secreted type of *β*1,6-N-acetylglucosaminyltransferase V (GnT-V) induces tumor angiogenesis without mediation of glycosylation. A novel function of GnT-V distinct from the original glycosyltransferase activity. *Journal of Biological Chemistry*.

[26] Filmus J, Selleck SB (2001). Glypicans: proteoglycans with a surprise. *Journal of Clinical Investigation*.

[27] Capurro M, Wanless IR, Sherman M (2003). Glypican-3: a novel serum and histochemical marker for hepatocellular carcinoma. *Gastroenterology*.

[28] Capurro MI, Xiang Y-Y, Lobe C, Filmus J (2005). Glypican-3 promotes the growth of hepatocellular carcinoma by stimulating canonical Wnt signaling. *Cancer Research*.

[29] Yamanaka K, Ito Y, Okuyama N (2008). Immunohistochemical study of glypican 3 in thyroid cancer. *Oncology*.

[30] Bartolazzi A, Orlandi F, Saggiorato E (2008). Galectin-3-expression analysis in the surgical selection of follicular thyroid nodules with indeterminate fine-needle aspiration cytology: a prospective multicentre study. *The Lancet Oncology*.

[31] Inohara H, Segawa T, Miyauchi A (2008). Cytoplasmic and serum galectin-3 in diagnosis of thyroid malignancies. *Biochemical and Biophysical Research Communications*.

[32] Medvedová L, Farkaš R (2004). Hormonal control of protein glycosylation: role of steroids and related lipophilic ligands. *Endocrine Regulations*.

[33] Varki A (2008). Sialic acids in human health and disease. *Trends in Molecular Medicine*.

[34] Babál P, Janega P, Černá A, Kholová I, Brabencová E (2006). Neoplastic transformation of the thyroid gland is accompanied by changes in cellular sialylation. *Acta Histochemica*.

